# Dynapenic Obesity and Prevalence of Type 2 Diabetes in Middle-Aged Japanese Men

**DOI:** 10.2188/jea.JE20140256

**Published:** 2015-11-05

**Authors:** Ryoko Kawakami, Susumu S. Sawada, I-Min Lee, Munehiro Matsushita, Yuko Gando, Takashi Okamoto, Koji Tsukamoto, Mitsuru Higuchi, Motohiko Miyachi, Steven N. Blair

**Affiliations:** 1 Graduate School of Sport Sciences, Waseda University, Tokorozawa, Saitama, Japan; 1 早稲田大学大学院 スポーツ科学研究科; 2 Department of Health Promotion and Exercise, National Institute of Health and Nutrition, Tokyo, Japan; 2 国立健康・栄養研究所 健康増進研究部; 3 Research Fellow of Japan Society for the Promotion of Science, Tokyo, Japan; 3 日本学術振興会 特別研究員; 4 Harvard Medical School, Boston, MA, USA; 4 ハーバード大学医学大学院; 5 Health Promotion Center, Tokyo Gas Co., Ltd., Tokyo, Japan; 5 東京ガス株式会社 安全健康・福利室; 6 Faculty of Sport Sciences, Waseda University, Tokorozawa, Saitama, Japan; 6 早稲田大学 スポーツ科学学術院; 7 University of South Carolina, Columbia, SC, USA; 7 サウスカロライナ大学

**Keywords:** muscle strength, hand strength, body mass index, hyperglycemia, dynapenia

## Abstract

**Background:**

The independent and combined associations of muscle strength and obesity on the prevalence of type 2 diabetes in Japanese men remain unclear.

**Methods:**

Hand grip strength was cross-sectionally evaluated between 2011 and 2013 to assess muscle strength in 5039 male workers aged 40 to 64 years. Weight and height were measured, and overweight/obesity was defined as a body mass index ≥25 kg/m^2^. The prevalence of type 2 diabetes, defined as fasting plasma glucose ≥126 mg/dL and/or hemoglobin A1c ≥6.5% and/or self-reported physician-diagnosed diabetes, was evaluated. Odds ratios (OR) and 95% confidence intervals (95% CI) for the prevalence of type 2 diabetes were obtained using a logistic regression model.

**Results:**

In total, 611 participants had type 2 diabetes, and 1763 participants were overweight/obese. After adjustment for covariates, we found an inverse association between muscle strength and the prevalence of type 2 diabetes (*P* for trend <0.01). In addition, when the analyses were stratified by obesity status, the multivariable-adjusted OR per 2-standard-deviation increase in muscle strength was 0.64 (95% CI, 0.49–0.83) in the overweight/obese group, compared to a weaker relationship in the normal-weight group (OR 0.79 per 2-standard-deviation increase; 95% CI, 0.60–1.06).

**Conclusions:**

Dynapenia, an age-related decrease in muscle strength, is associated with increased prevalence of type 2 diabetes, and this relationship is stronger in overweight/obese middle-aged Japanese men than in normal-weight men.

## Abstract (ja)

**【背景】:**

加齢に伴う筋力低下をダイナペニアという。本研究は、2型糖尿病有病率に対する筋力と肥満の独立および複合の関連を横断的に検討することを目的とした。

**【方法】:**

対象者は、2011年から2013年の間に握力の測定を行った40から64歳の男性労働者5039名であった。体重と身長の測定を行い、BMIが25kg/m^2^以上の者を過体重/肥満と判定した。2型糖尿病の判定は、空腹時血糖126mg/dl以上、ヘモグロビンA1c6.5%以上、医師による2型糖尿病の診断（自記式問診票）のいずれか一つでも該当した場合とした。ロジスティック回帰モデルを用いて2型糖尿病有病者のオッズ比および95%信頼区間を求めた。

**【結果】:**

2型糖尿病有病者は611名、過体重/肥満者は1763名であった。共変量を調整し検討したところ、筋力と2型糖尿病有病率との間に負の関係が認められた（トレンド検定 P<0.01）。肥満状況で層別解析した結果、過体重/肥満者において筋力の2標準偏差増加あたりの多変量調整オッズ比は0.64（95%信頼区間0.49-0.83）と有意な関連が認められたのに対して、正常者では関連がみられなかった（2標準偏差増加あたりのオッズ比 0.79、95%信頼区間0.60-1.06）。

**【結論】:**

日本人中年男性において、加齢に伴う筋力の低下（ダイナペニア）は2型糖尿病の高い有病率と関連し、この関連性は過体重/肥満者において強く認められた。

## INTRODUCTION

The number of people with type 2 diabetes is increasing globally, with the International Diabetes Federation reporting that 382 million people worldwide suffered from diabetes in 2013; this number is predicted to rise to 592 million by 2035.^1^ It has been established that obesity is a common risk factor for type 2 diabetes.^2^^,^^3^ However, through lifestyle modifications, which include a healthy diet and regular physical activity, type 2 diabetes can be delayed and even prevented.^4^

Dynapenia is the age-related decrease in muscle strength.^5^ This decline in muscle strength begins in midlife and accelerates with age.^6^^–^^8^ During this same time period, the percentage of body fat increases with age until around 80 years of age.^9^

Sayer et al reported an inverse dose-response relationship between muscle strength and insulin resistance as well as 2-hour glucose levels in an oral glucose tolerance test.^10^ Studies have shown that engaging in muscle-strengthening activities is associated with a reduced risk of developing type 2 diabetes.^11^

Individuals with sarcopenia (ie, low muscle mass) along with obesity have a higher degree of insulin resistance and a higher prevalence of dysglycemia than obese individuals without sarcopenia.^12^ A recent study also demonstrated that people with dynapenic abdominal obesity had a higher prevalence of type 2 diabetes than those with neither dynapenia nor abdominal obesity.^13^ However, a limited number of epidemiologic studies have examined the relationship between dynapenic obesity and the prevalence of type 2 diabetes. We hypothesized that the coexistence of dynapenia and obesity may synergistically induce the development of type 2 diabetes. Therefore, we cross-sectionally examined the independent and combined associations of muscle strength and obesity on the prevalence of type 2 diabetes in middle-aged Japanese men.

## METHODS

### Participants

Participants were male workers of a gas company in Tokyo, Japan. All workers receive annual health checkups, in accordance with the Industrial Safety and Health Law. A total of 5039 male workers aged 40 to 64 years who completed all measurements, including muscle strength testing and assessment of confounders, were included in the present analysis (89.5% of all male workers in this age group). Data were collected between October 2011 and March 2013. Female workers were excluded due to the small sample size. This study was approved by the Ethics Review Board of the National Institute of Health and Nutrition, and all participants provided written informed consent.

### Clinical examinations

The annual health checkup, including a blood test and measurement of height, body weight, and blood pressure, was conducted in the morning after an overnight fast. Height and body weight were measured to the nearest 0.1 cm and 0.1 kg, respectively. Body mass index (BMI) was calculated as the body weight divided by the square of the height (kg/m^2^). Blood pressure was measured using a standard auscultatory method. Medical history, cigarette smoking, alcohol intake, and family history of diabetes were assessed using a self-administered questionnaire. Family history of diabetes was defined as the known presence of family members with diabetes in any of three generations. Overweight/obesity was defined as a BMI ≥25 kg/m^2^, based on the World Health Organization criteria.

### Muscle strength measurement

Previous studies have shown a high correlation between hand grip strength and other measurements of muscle strength, such as knee extension and flexion.^14^^–^^16^ Therefore, we used hand grip strength to define overall muscle strength. Hand grip strength was measured using a digital hand dynamometer (T.K.K.5401; Takei Scientific Instruments Co., Ltd., Japan) to the nearest 0.1 kg. The participants were instructed to stand and hold the dynamometer at maximal capacity with the elbow straight. One trial for each hand was performed, and the average of both hands was taken and used for the present analyses. The participants were divided into quartiles depending on age-specific (40–49, 50–59, and 60–64 years) muscle strength. The cut-off values for each age group are shown in Table 1. In the analysis stratified by obesity status (BMI ≥25 kg/m^2^ [overweight/obese] or <25 kg/m^2^ [normal weight]), the participants were divided into quartiles depending on age-specific muscle strength for each obesity status.

**Table 1.** Age-specific cut-off values for muscle strength based on quartiles

Muscle strength (Hand grip strength, kg)	Age		

	40–49 years	50–59 years	60–64 years
Q1 (Lowest)	≤37.0	≤35.3	≤33.7
Q2	37.1–40.8	35.4–38.8	33.8–37.6
Q3	40.9–44.9	38.9–42.7	37.7–41.0
Q4 (Highest)	≥45.0	≥42.8	≥41.1

### Assessment of type 2 diabetes

We estimated the prevalence of type 2 diabetes, defined as fasting plasma glucose ≥126 mg/dL (7.0 mmol/L) and/or hemoglobin A1c (HbA1c; NGSP) ≥6.5% and/or self-reported physician-diagnosed diabetes (under treatment or no treatment but being followed). The criteria for fasting plasma glucose and HbA1c in the diagnosis of type 2 diabetes were based on the diagnostic guidelines of the American Diabetes Association^17^ and the Japan Diabetes Society.^18^

### Statistical analysis

We compared characteristics of participants by diabetes status and muscle strength categories using Student’s *t*-tests or one-way ANOVA for continuous variables and chi-squared tests for categorical variables, as appropriate. We used logistic regression models to estimate odds ratios (ORs) and 95% confidence intervals (CIs) for the prevalence of type 2 diabetes, adjusted for age (continuous variable), systolic blood pressure (continuous variable), cigarette smoking (never, former, or current smoker), alcohol intake (drinker or non-drinker), family history of diabetes (yes or no), and BMI (continuous variable) or hand grip strength (continuous variable) in a multivariable model. The ORs for the prevalence of type 2 diabetes per 2-standard-deviation (SD) change were also calculated. A two-tailed *P* value less than 0.05 was considered statistically significant. All statistical analyses were performed with SPSS Statistics version 22 for Windows (IBM Japan, Tokyo, Japan).

## RESULTS

Of the 5039 participants, 611 (12.1%) had type 2 diabetes, and 1763 (35.0%) were overweight/obese. The mean age of the participants was 51 years (range, 40–64 years), and mean hand grip strength was 39.7 kg (SD, 6.0 kg).

Table 2 shows the characteristics of the participants according to diabetes status. Individuals with type 2 diabetes were older and had higher weight, BMI, systolic blood pressure, and diastolic blood pressure than those without type 2 diabetes. The participants with type 2 diabetes were also more likely to be smokers and to have a family history of diabetes. Furthermore, hand grip strength was lower in participants with type 2 diabetes than in those without type 2 diabetes.

**Table 2.** Characteristics according to diabetes status

	Total	Without diabetes	With diabetes	*P* value
*n*	5039	4428	611	
Age, years	51 (7)	51 (7)	55 (6)	<0.001
Height, cm	170.7 (5.8)	170.8 (5.8)	169.5 (5.6)	<0.001
Body weight, kg	70.6 (10.6)	70.1 (10.2)	74.4 (12.8)	<0.001
Body mass index, kg/m^2^	24.2 (3.3)	24.0 (3.1)	25.9 (4.1)	<0.001
Plasma glucose, mg/dL	103.9 (20.2)	98.6 (8.9)	142.4 (33.4)	<0.001
HbA1c, %	5.7 (0.7)	5.5 (0.3)	7.1 (1.1)	<0.001
Systolic blood pressure, mm Hg	126.9 (18.9)	125.9 (18.5)	134.2 (20.0)	<0.001
Diastolic blood pressure, mm Hg	81.2 (12.0)	80.7 (11.9)	84.8 (11.9)	<0.001
Hand grip strength, kg	39.7 (6.0)	39.9 (6.0)	38.8 (5.9)	<0.001
Plasma glucose ≥126 mg/dL, %	8.6	0	70.5	—
HbA1c ≥6.5%, %	9.3	0	76.9	—
Self-reported physician-diagnosed diabetes, %	7.2	0	59.7	—
Smokers, %	35.4	34.0	45.5	<0.001
Drinkers, %	85.6	86.3	80.4	<0.001
Family history of diabetes, %	23.6	21.1	41.9	<0.001

Data are express as mean (standard deviation) or percentages of participants.

Table 3 shows the characteristics of the participants according to muscle strength categories. Men in the lowest muscle strength group also had the lowest levels of BMI, systolic blood pressure, diastolic blood pressure, and alcohol consumption.

**Table 3.** Characteristics according to muscle strength categories

	Muscle strength				*P* value

	Q1 (Lowest)	Q2	Q3	Q4 (Highest)	
*n*	1266	1271	1253	1249	
Hand grip strength, kg	32.5 (3.0)	37.8 (1.5)	41.5 (1.7)	47.3 (3.7)	<0.001
Age, years	51 (7)	51 (7)	51 (7)	51 (7)	0.317
Height, cm	168.3 (5.6)	169.9 (5.5)	171.3 (5.4)	173.4 (5.6)	<0.001
Body weight, kg	66.3 (9.6)	69.3 (10.0)	71.4 (10.0)	75.6 (10.7)	<0.001
Body mass index, kg/m^2^	23.4 (3.2)	24.0 (3.3)	24.3 (3.1)	25.1 (3.2)	<0.001
Plasma glucose, mg/dL	103.4 (21.3)	104.6 (21.5)	103.8 (19.4)	103.9 (18.4)	0.561
HbA1c, %	5.7 (0.7)	5.7 (0.8)	5.6 (0.7)	5.6 (0.6)	0.080
Systolic blood pressure, mm Hg	124.3 (18.6)	126.7 (19.4)	127.6 (18.6)	129.2 (18.6)	<0.001
Diastolic blood pressure, mm Hg	79.7 (11.9)	80.9 (12.2)	81.5 (11.8)	82.7 (11.7)	<0.001
Smokers, %	34.0	34.5	38.0	35.1	0.162
Drinkers, %	81.9	86.9	86.8	86.7	<0.001
Family history of diabetes, %	22.4	24.3	22.9	24.8	0.421

Data are express as mean (standard deviation) or percentages of participants.

Table 4 shows the independent associations of either muscle strength or obesity with the prevalence of type 2 diabetes. In the model adjusted only for age, there was no association between muscle strength and the prevalence of type 2 diabetes (*P* for trend = 0.36). However, after additional adjustment for systolic blood pressure, cigarette smoking, alcohol intake, family history of diabetes, and BMI (model 2), there was an inverse association between muscle strength and prevalence of type 2 diabetes (*P* for trend <0.01), and the multivariable-adjusted OR per 2-SD (12.0 kg) increase in hand grip strength was 0.69 (95% CI, 0.57–0.84). We also identified a positive association between BMI and prevalence of type 2 diabetes (per 2-SD decrease, OR 0.35; 95% CI, 0.29–0.42). These data suggest that lower muscle strength and higher BMI are associated with a higher prevalence of type 2 diabetes.

**Table 4.** Odds ratios for prevalence of type 2 diabetes according to muscle strength categories and obesity status

	*n*	Number of cases	Number of cases (per 1000 persons)	Age-adjusted OR (95% CI)	Model 1^a^ OR (95% CI)	Model 2^b^ OR (95% CI)
Muscle strength						
Q1 (Lowest)	1266	157	124	1.00 (Reference)	1.00 (Reference)	1.00 (Reference)
Q2	1271	170	134	1.10 (0.87–1.40)	1.06 (0.83–1.36)	0.99 (0.77–1.27)
Q3	1253	143	114	0.92 (0.72–1.18)	0.86 (0.67–1.11)	0.79 (0.61–1.02)
Q4 (Highest)	1249	141	113	0.94 (0.73–1.20)	0.83 (0.64–1.08)	0.68 (0.52–0.88)
*P* for trend				0.355	0.066	0.001
per 2-SD increase (12.0 kg)				0.91 (0.76–1.10)	0.83 (0.69–1.00)	0.69 (0.57–0.84)

Obesity						
Overweight/obese (BMI ≥25 kg/m^2^)	1763	331	188	1.00 (Reference)	1.00 (Reference)	1.00 (Reference)
Normal weight (BMI <25 kg/m^2^)	3276	280	85	0.39 (0.33–0.47)	0.45 (0.38–0.55)	0.44 (0.36–0.53)
per 2-SD decrease (6.6 kg/m^2^)				0.32 (0.27–0.38)	0.37 (0.31–0.44)	0.35 (0.29–0.42)

BMI, body mass index; CI, confidence interval; OR, odds ratio; Q, quartile; SD, standard deviation.

^a^Model 1: Adjusted for age, systolic blood pressure, cigarette smoking, alcohol intake, and family history of diabetes.

^b^Model 2: Adjusted for Model 1 covariates plus BMI (for muscle strength categories) or hand grip strength (for obesity status).

We went on to investigate the odds of type 2 diabetes according to muscle strength categories in analyses stratified by obesity status (BMI ≥25 kg/m^2^ or <25 kg/m^2^) (Table 5). Using the lowest muscle strength group (Q1) as a reference and adjusting for age, systolic blood pressure, cigarette smoking, alcohol intake, family history of diabetes, and BMI (model 2), the ORs for the second, third, and fourth quartiles of muscle strength were 0.77 (95% CI, 0.54–1.10), 0.70 (95% CI, 0.49–0.99), and 0.54 (95% CI, 0.38–0.79), respectively (*P* for trend <0.01). The multivariable-adjusted OR per 2-SD (12.0 kg) increase in hand grip strength was 0.64 (95% CI, 0.49–0.83) in the overweight/obese group; however, the relationship was weaker in the normal weight group (per 2-SD increase, OR 0.79; 95% CI, 0.60–1.06; *P* for trend = 0.43). There was no interaction between muscle strength (continuous variable) and BMI (continuous variable) (*P* = 0.89).

**Table 5.** Odds ratios for the prevalence of type 2 diabetes according to muscle strength categories in analysis stratified by obesity status

	*n*	Number of cases	Number of cases (per 1000 persons)	Age-adjusted OR (95% CI)	Model 1^a^ OR (95% CI)	Model 2^b^ OR (95% CI)
Overweight/obese (BMI ≥25 kg/m^2^), *n* = 1763						
Q1 (Lowest)	444	102	230	1.00 (Reference)	1.00 (Reference)	1.00 (Reference)
Q2	446	84	188	0.78 (0.56–1.09)	0.82 (0.58–1.15)	0.77 (0.54–1.10)
Q3	437	77	176	0.72 (0.51–1.01)	0.72 (0.51–1.03)	0.70 (0.49–0.99)
Q4 (Highest)	436	68	156	0.64 (0.45–0.90)	0.60 (0.42–0.86)	0.54 (0.38–0.79)
*P* for trend				0.009	0.004	0.001
per 2-SD increase (12.0 kg)				0.73 (0.57–0.94)	0.69 (0.53–0.89)	0.64 (0.49–0.83)

Normal weight (BMI <25 kg/m^2^), *n* = 3276						
Q1 (Lowest)	834	70	84	1.00 (Reference)	1.00 (Reference)	1.00 (Reference)
Q2	810	78	96	1.20 (0.84–1.69)	1.19 (0.83–1.71)	1.17 (0.81–1.68)
Q3	826	68	82	1.02 (0.71–1.45)	0.93 (0.64–1.35)	0.91 (0.62–1.32)
Q4 (Highest)	806	64	79	1.01 (0.70–1.46)	0.98 (0.67–1.43)	0.93 (0.63–1.36)
*P* for trend				0.835	0.603	0.428
per 2-SD increase (12.0 kg)				0.88 (0.67–1.16)	0.83 (0.63–1.10)	0.79 (0.60–1.06)

BMI, body mass index; CI, confidence interval; OR, odds ratio; Q, quartile; SD, standard deviation.

^a^Model 1: Adjusted for age, systolic blood pressure, cigarette smoking, alcohol intake, and family history of diabetes.

^b^Model 2: Adjusted for Model 1 covariates plus BMI.

Figure illustrates the results of multivariable-adjusted ORs and 95% CIs for the prevalence of type 2 diabetes according to combined muscle strength categories and obesity status (BMI ≥25 kg/m^2^ or <25 kg/m^2^). Using the lowest muscle strength group (Q1) with overweight/obese men as the reference and adjusting for age, systolic blood pressure, cigarette smoking, alcohol intake, and family history of diabetes, the ORs from the second, third, and fourth quartiles of muscle strength with overweight/obese men were 0.89 (95% CI, 0.62–1.29), 0.65 (95% CI, 0.44–0.95), and 0.61 (95% CI, 0.43–0.88), respectively (*P* for trend <0.01). In contrast, this relationship was not observed in the normal weight group (*P* for trend = 0.53). These data suggest that lower muscle strength combined with obesity is associated with a higher prevalence of type 2 diabetes.

**Figure.**
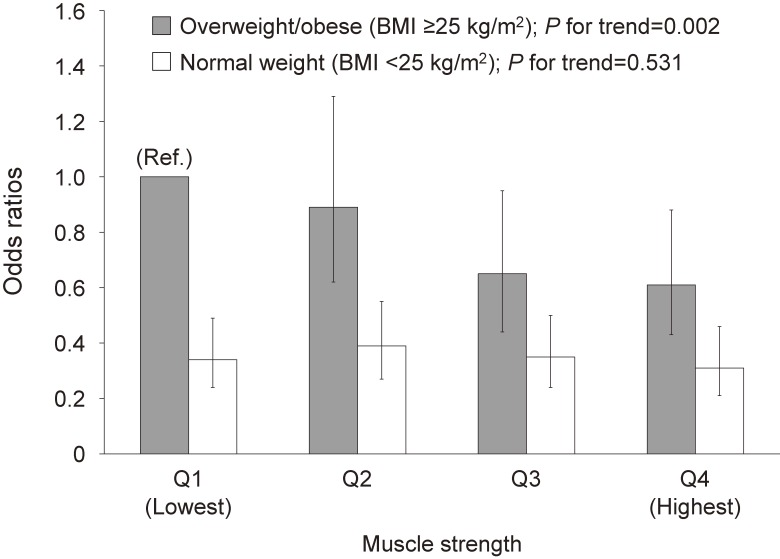
Multivariable-adjusted odds ratios and 95% confidence intervals for prevalence of type 2 diabetes, according to combined muscle strength categories and obesity status, after adjustment for age, systolic blood pressure, cigarette smoking, alcohol intake, and family history of diabetes.

## DISCUSSION

In this cross-sectional study of Japanese men, we investigated the independent and combined associations of muscle strength and obesity on the prevalence of type 2 diabetes. We confirmed that the prevalence of type 2 diabetes was positively associated with BMI and was inversely associated with muscle strength (Table 4). Interestingly, we found that muscle strength appeared to be inversely associated with prevalence of type 2 diabetes only in overweight/obese men (Table 5 and Figure).

Our data, as well as the data from numerous other studies,^10^^,^^11^^,^^19^^–^^26^ support the hypothesis that muscle strength is a contributing factor to type 2 diabetes. Muscle strength has been shown to be inversely associated with insulin resistance.^10^^,^^19^^–^^21^ Sayer et al revealed a significant inverse dose-response relationship between hand grip strength and insulin resistance, as well as the 2-hour glucose level in an oral glucose tolerance test.^10^ Other studies have also reported that people with diabetes have lower muscle strength than those without diabetes.^22^^,^^23^ Engaging in muscle-strengthening activities is associated with a reduced risk of developing type 2 diabetes among women from the Nurses’ Health Study and Nurses’ Health Study II.^11^ According to the National Health and Nutrition Examination Survey (NHANES), muscle-strengthening activities may also have favorable effects on impaired fasting glucose, insulin sensitivity, and waist circumference among men and women.^24^^,^^25^ The Aerobics Center Longitudinal Study demonstrated that muscle strength, assessed by the one-repetition maximal measures for bench and leg press, was inversely associated with incidence of metabolic syndrome in men.^26^ These findings are consistent with our data and conclusions that low muscle strength (ie, dynapenia) is associated with a higher prevalence of type 2 diabetes.

In the present study, overweight/obese men in the lowest quartile of hand grip strength demonstrated a significantly higher prevalence of type 2 diabetes than men with a normal weight in the highest quartile of hand grip strength (Figure), showing that dynapenic obesity is robustly associated with type 2 diabetes. We performed an analysis stratified by obesity status, which showed that muscle strength was inversely associated with the prevalence of type 2 diabetes in overweight/obese men; this relationship was not observed in men with a normal weight (Table 5). The association of sarcopenia with obesity and insulin resistance has been investigated in several studies.^12^^,^^27^ Using cross-sectional data from the NHANES III, Srikanthan et al reported that patients with sarcopenic obesity have a higher degree of insulin resistance and a higher prevalence of dysglycemia than obese patients without sarcopenia.^12^ In a prospective cohort study, Stephan et al showed that dynapenic abdominal obesity, as determined by low hand grip strength with high waist circumference, was modestly associated with an increased incidence of cardiovascular disease in older adults; in contrast, there was no relationship between sarcopenia, as determined by low muscle mass, with abdominal obesity and cardiovascular disease.^28^ Using NHANES data, a recent cross-sectional study demonstrated that people with dynapenic abdominal obesity had a higher prevalence of type 2 diabetes (assessed by a self-reported questionnaire) than those with neither dynapenia nor abdominal obesity.^13^ Wander et al reported that higher hand grip strength was associated with a lower incidence of type 2 diabetes during a 10-year follow-up in Japanese-American men and women.^29^ Taken together, these data support our finding that muscle strength and obesity are dependent factors contributing to the prevalence of type 2 diabetes.

Wander et al identified a significant association between hand grip strength and BMI, which was attenuated at higher levels of BMI^29^; however, this association between hand grip strength and BMI was not observed in our study. The reason for this discrepancy is unclear but may be due to differences in the characteristics of the participants. Wander et al included both men (*n* = 209) and women (*n* = 185) in their analysis, while our study only included men. Men’s hand grip strength tends to be substantially higher than women’s on average, so our mean hand grip strength was much higher than that in Wander et al study’s (39.7 kg versus 22.9 kg). This relatively large difference may be the source of the discrepancy in findings between these two studies.

Although the mechanisms for the association between dynapenic obesity and the prevalence of type 2 diabetes are unknown, the association between obesity and insulin resistance may be related to inflammatory cytokines. Schrager et al found that dynapenic obesity was associated with high levels of interleukin-6 and C-reactive protein.^30^ Moreover, they found that dynapenia and obesity had an additive effect on the levels of these inflammatory cytokines. Therefore, proinflammatory cytokines might mediate the reduction in muscle strength and further promote insulin resistance. Additionally, skeletal muscle is a major tissue for glucose uptake and utilization, and muscle strength training has been shown to increase the protein content of glucose transporter 4.^31^^,^^32^ These findings suggest that the coexistence of dynapenia and obesity may synergistically induce the development of type 2 diabetes.

This study had several limitations. First, this study had a cross-sectional design. Therefore, further longitudinal studies are necessary to establish a causal relationship between dynapenic obesity and type 2 diabetes. Type 2 diabetes is associated with accelerated loss of muscle strength and muscle mass,^33^^,^^34^ so our results may merely be reflecting a consequence of the disease. However, Park et al reported that the rapid loss of thigh muscle mass was observed in women with diabetes but not in men.^33^ Also, no difference in loss of hand grip strength has been observed between those with and without diabetes over a 3-year period.^34^ Second, in the present study, hand grip strength was measured with only one trial for each hand. However, Watanabe et al tested the same dynamometer used in our study and reported that there was no difference between first and second trials in men.^35^ Hamilton et al showed high reliability with one trial (intraclass correlation coefficients ≥0.93) compared with the mean of two or three trials.^36^ Finally, the participants were middle-aged (40–64 years) male workers, so it is unclear whether the investigated associations also exist among women and older men. Further, all participants were employees of a single, urban company and may not be representative of all Japanese men.

In conclusion, dynapenia, the age-related decrease in muscle strength, is associated with an increased prevalence of type 2 diabetes, and this relationship is stronger in overweight/obese middle-aged Japanese men than in those of normal weight.

## ONLINE ONLY MATERIAL

Abstract in Japanese.
