# Supplementary material for: Dynapenic Obesity and Prevalence of Type 2 Diabetes in Middle-Aged Japanese Men
Source: J Epidemiol. 2015 Nov 5;25(10):656–62. doi: 10.2188/jea.JE20140256 (PMC4626395; doi:10.2188/jea.JE20140256)
Supplement: Abstract in Japanese. [file je-25-656-s001.pdf]

日本人中年男性におけるダイナペニア肥満と 2 型糖尿病有病率との関連

川上諒子 1,2,3、澤田亨 2、I-Min Lee 4、松下宗洋 1,2、丸藤祐子 2、岡本隆史 5、塚本浩二 5、樋口満 6、宮地元彦 2、Steven N. Blair 7

1 早稲田大学大学院 スポーツ科学研究科、2 国立健康・栄養研究所 健康増進研究部、3 日本学術振興会 特別研究員、4 ハーバード大学医学大学院、5 東京ガス株式会社 安全健康・福利室、6 早稲田大学 スポーツ科学学術院、7 サウスカロライナ大学

【背景】加齢に伴う筋力低下をダイナペニアという。本研究は、2 型糖尿病有病率に対する筋力と肥満の独立および複合の関連を横断的に検討することを目的とした。
【方法】対象者は、2011 年から 2013 年の間に握力の測定を行った 40 から 64 歳の男性労働者 5039 名であった。体重と身長の測定を行い、BMI が 25kg/m^2 以上の者を過体重/肥満と判定した。2 型糖尿病の判定は、空腹時血糖 126mg/dl 以上、ヘモグロビン A1c6.5%以上、医師による 2 型糖尿病の診断（自記式問診票）のいずれか一つでも該当した場合とした。ロジスティック回帰モデルを用いて 2 型糖尿病有病者のオッズ比および 95%信頼区間を求めた。
【結果】2 型糖尿病有病者は 611 名、過体重/肥満者は 1763 名であった。共変量を調整し検討したところ、筋力と 2 型糖尿病有病率との間に負の関係が認められた（トレンド検定 P<0.01）。肥満状況で層別解析した結果、過体重/肥満者において筋力の 2 標準偏差増加あたりの多変量調整オッズ比は 0.64（95%信頼区間 0.49-0.83）と有意な関連が認められたのに対して、正常者では関連がみられなかった（2 標準偏差増加あたりのオッズ比 0.79、95%信頼区間 0.60-1.06）。
【結論】日本人中年男性において、加齢に伴う筋力の低下（ダイナペニア）は 2 型糖尿病の高い有病率と関連し、この関連性は過体重/肥満者において強く認められた。

キーワード：筋力、握力、BMI、高血糖、ダイナペニア
